# Global research status and hotspot analysis of meniscal root tears based on the WOS database

**DOI:** 10.3389/fsurg.2022.944566

**Published:** 2022-09-09

**Authors:** Yifan Wang, Chen Huang, Yansong Qi, Huricha Bao, Yongsheng Xu

**Affiliations:** ^1^ Department of Orthopedics (Sports Medicine Center), Inner Mongolia People's Hospital, Hohhot, China; ^2^ Graduate School, Inner Mongolia Medical University, Hohhot, China; ^3^ Center of Basic Medical Research, Peking University Third Hospital, Beijing, China

**Keywords:** meniscus, root tear, research status, research hotspots, CiteSpace, VOSviewer, web of science

## Abstract

**Background:**

Meniscal root tears are one of the common diseases in the field of orthopedics and sports medicine today and are the subject of many current investigative efforts.

**Purpose:**

This study aims to identify and evaluate the global trends, hotspots and frontiers in meniscal root tear research using bibliometric analysis.

**Methods:**

A bibliometric analysis of research findings related to meniscal root tears over the past three decades was performed. CiteSpace was used to conduct document co-citation and cluster analyses on the collected data. The research was conducted based on the following factors: country and institution distribution, chronological distribution, source journal analysis, keyword co-occurrence analysis, and reference co-citation analysis.

**Results:**

A total of 626 research articles on meniscal root tears in English published from 1989 to 2021 were obtained. There was a significant upward trend in the total number of scientific publications over the past decades, especially in 2015–2020. The most productive countries, institutions, journals and authors are the **USA, STEADMAN PHILIPPON, *KNEE SURGERY SPORTS TRAUMATOLOGY ARTHROSCOPY***, and **LAPRADE RF**. North America and East Asia made outstanding contributions to the research on meniscal root tears, but cooperation and exchanges between countries and institutions were not close enough. A total of 9 clusters were obtained from the citation analysis, and 8 clusters were obtained from the keyword analysis. The main keywords that ranked first were posterior root tear, medial meniscus, menisci tibial, and ACL reconstruction, and these clusters combined with the corresponding emergence reflected the current status of research at different times.

**Conclusion:**

Research in this field over the past 32 years has gone through a phase of exploration in the understanding of the anatomy of the meniscal root and the diagnosis of this disease and a phase of development with in-depth biomechanical studies and improved and innovative surgical techniques. The current research focuses on the innovation of meniscal root tear repair techniques, the long-term efficacy of surgery, the variability in the efficacy of different surgical techniques, and surgical strategies for combined injuries. There will be more breakthroughs in surgical techniques, surgical equipment and surgical materials to resolve meniscal root tears.

## Introduction

The knee meniscus is a fibrocartilaginous structure located in the tibiofemoral joint whose main role is to enhance knee stability, distribute pressure loads evenly, and reduce articular cartilage loading while contributing to proprioception, cartilage nutrition, and lubrication. Approximately 40%–70% of the load through the knee joint is borne by the meniscus, whose mechanical load-absorbing capacity converts axial loads into circumferential stresses (1, 2). The meniscal root also plays an important role in maintaining knee joint stability. The integrity of the meniscal root, the attachment point of the medial and lateral meniscus in the intercondylar region of the tibial plateau, is essential to maintain proper knee kinematics and prevent degenerative changes in the joint. In 1991, Pagnani et al. first described meniscal root tears, and the importance of meniscal root integrity has received increasing attention over the past 30 years (3). Meniscal extrusion increases the stress on the cartilage by reducing the contact surface, which leads to circumferential hoop stress and dissipation injury, causing biomechanical changes and accelerating the degenerative process (1). A tear within 1 cm of the bony attachment point of the meniscus or an avulsion injury at the tibial attachment of the meniscus root is defined as a meniscus root tear (MRT). LaPrade et al. classified MRT tears into five types based on tear morphology: type 1 (7%), partial stable root tears; type 2 (67.6%), complete radial tears within 9 mm from the root attachment, which is the most common type of tear, including type 2A (38%): 0–3 mm, type 2B (16.9%): 3–6 mm, and type 2C (12.7%): 6–9 mm; type 3 (5.6%), bucket-handle tears with root detachment; type 4 (9.9%), complex oblique or longitudinal tears extending into the root attachment; and type 5 (9.9%), bony avulsion fractures of the root attachments (4). Meniscal root tears can occur chronically in the degenerative knee or after acute trauma. Traditionally, the posterior root of the meniscus is subjected to more load than the anterior root and is more susceptible to injury, especially when the knee is flexed at 90°. Medial meniscus posterior root tears (MMPRTs) are the most common lesion, with a prevalence of 10%–21% in meniscal surgery. The posterior root of the medial meniscus is more prone to lesions than the posterior lateral root due to its reduced mobility and high loading (5–7). The incidence of MMPRTs is increased in parts of the world where a squatting position is common (8). Increased BMI, female sex, low activity level, varus mechanical axis, and some unknown intrinsic factors have been associated with an increased risk of MMPRTs (9, 10). Lateral meniscal posterior root tears (LMPRTs) appear to be more frequently associated with knee sprains. According to De Smet et al. (11), LMPRTs could be identified in 8% of patients with ACL injury and 0.8% of patients with intact ACL.

The clinical diagnosis of meniscal root tears is difficult, and the differential diagnosis of posterior root tears in particular is extremely challenging. The symptoms of the lateral side in acute injuries could not be present or hidden by associated masked ACL tears. In chronic cases, patients may complain of posterior knee pain, especially in extreme flexion (12). MRI is currently considered the gold standard for the diagnosis of meniscal root tears, and the ghost sign and meniscal compression manifestations have been described as characteristic of meniscal root tears (13). The main treatment modalities for meniscal root injuries include conservative management, meniscectomy and repair. The choice of treatment modality should be based on the general condition of the patient, the type of root injury, and the overall condition of the cartilage, but most investigators prefer early meniscal root repair for root injuries because it can approximate the biomechanical restoration of the meniscus and stop the progression of osteoarthritis (14–17). The biomechanics of meniscal root injuries, clinical diagnosis, and surgical repair techniques have been extensively studied in recent decades. An overview of the contents related to meniscal root injuries in different regions of the world is also important and helpful for researchers to identify research trends, track research hotspots, and determine the direction of research in the coming years.

Bibliometrics is a popular and effective method that integrates philology, statistics, and mathematics in a comprehensive discipline focusing on the number of publications. Bibliometrics is widely used to research frontiers and explore trends in various research fields (18). Scientific mapping is a new scientific research method that can explore the source of knowledge and its development pattern, and can express the relationship and evolution pattern of knowledge structure in related fields in the form of graphics (19). CiteSpace is a web-based Java data analysis and visualization application for mapping knowledge of the scientific literature and showing trends in technical fields (18, 20). This study used CiteSpace software to conduct in-depth mining and analysis of the literature related to meniscal root injury from 1989 to 2021, to visualize the development process, research hotspots and cutting-edge trends in this field, and to provide a comprehensive and scientific guide for future research by understanding the current status of meniscal root injury research.

## Methods

### Data sources

A search for articles related to meniscal root tears was conducted in the Web of Science (WOS) Core Collection (SCI-EXPANDED, SSCI, A/HCI, CPCI-S, CPCI-SSH, ESCI, CCR-EXPANDED, and IC). The search formula was: # 1, [TS = (meniscus OR meniscal) OR TI = (meniscus OR meniscal) OR AB = (meniscus OR meniscal)] AND LANGUAGE: (English) AND DOCUMENT TYPES: (Article OR Review); # 2, [TS = (“root tear” OR “posterior horn tear” OR “root tears” OR “posterior horn tears” OR “avulsion”) OR TI = (“root tear” OR “posterior horn tear” OR “root tears” OR “posterior horn tears” OR “avulsion”) OR AB = (“root tear” OR “posterior horn tear” OR “root tears” OR “posterior horn tears” OR “avulsion”)] AND LANGUAGE: (English) AND DOCUMENT TYPES: (Article OR Review); # 3, “# 1” AND “# 2”. TS stands for “Topic Subject” search in the WOS search, which is a Boolean logic-based method of using subject words to search for data, allowing quick and easy access to a large amount of subject-related data. The search time was set from 1989 to 2021, and the data collection date was 4 August 2021. The retrieved results were extracted, filtered, compared, and weighted, resulting in 626 records. The search records were exported to CiteSpace for further analysis, and the search result documents were filtered and downloaded, each download including author, title, abstract, descriptor and bibliographic records.

### Inclusion and exclusion criteria

The inclusion criteria were as follows: (a) articles related to meniscal root tears, including clinical studies and conference articles and abstracts; (b) reviews of meniscal root tears; (c) articles published from 1989 to 2021; (d) articles retrieved from the WOS database; and (e) articles in English. The exclusion criteria were as follows: (a) letters, letters to the editor and errata documents; (b) irrelevant articles; (c) duplicate publications; (d) articles collected manually and by phone; (e) articles not formally published; and (f) non-English articles.

### Data analysis

CiteSpace 6.1. R2, and Microsoft Excel 2019 were used to analyze and visualize the literature on MRT research.

Co-citation analysis studies are based on the scientific research structure of citations and co-citations. If two references appear in the same bibliography, they are considered to have co-citations (21), and it is generally believed that the same cited literature has more or less similarity in subject matter, so the number of co-citations, i.e., co-citation strength, can measure the relevance of literature in terms of content. As a result, a co-citation network can be formed by the co-citation relationship between a group of documents, and the proximity of the nodes within the network can reflect the closeness of their subject contents. Due to the objectivity and scientific nature of the co-citation analysis method, its analysis has been extended from papers to authors, journals and disciplines. Keywords are the core summary of a paper, and the analysis of keywords in a paper can give a general understanding of the topic of the article (22). And keyword co-occurrence analysis is also derived from the concept of citation and co-citation in bibliometrics. CiteSpace software is well known for its powerful co-citation analysis of literature, which combines the dual nature and characteristics of “graph” and “spectrum”, focusing on tree graphs and lines to represent the strength of the relationship between each topic, by using CiteSpace to investigate the research results, disciplinary distribution, publication sources, active countries, institutions and researchers related to meniscal root injury in the past 30 years, it can help researchers who are new to the field to understand the development of the field, the top research teams and journals in the field, and to clarify the cooperation between organizations and personnel, so that they can choose their partners or The foundation is laid for the subsequent selection of partners or journals to be submitted. By using multivariate statistical methods such as cluster analysis and multidimensional scale analysis, we can summarize the research hotspots, cutting-edge knowledge and research dynamics in the field (23).

The parameters of CiteSpace were set as follows: time slicing (1989–2021), years per slice (1 year), term source (all selections), node type (1 type), selection criteria (top 50) and pruning method (pathfinder method), and visualization (static cluster view and display merged network). For each node (nodes in the graph represent node types such as author, institution, country, citation, keyword and other types), we analyzed node size (implying frequency of occurrence or citation), connectivity between nodes (representing collaborative, co-citation, or synergistic relationships), betweenness centrality (indicating key nodes in collaborative or inspired networks), burst detection (red ring), and focus of important nodes. The node size is proportional to the frequency of its type. The number of lines indicates the degree of connectivity between nodes. Betweenness centrality is used to quantify the importance of a node's position in the network. The higher the betweenness centrality is, the higher the number of connections through that node in the network. Nodes with betweenness centrality greater than 0.1 are usually marked with purple circles (24). Microsoft Excel was used to create tables and show annual country trends for publications and citations.

## Results and discussion

### Temporal trends of distribution

**Figure 1:**
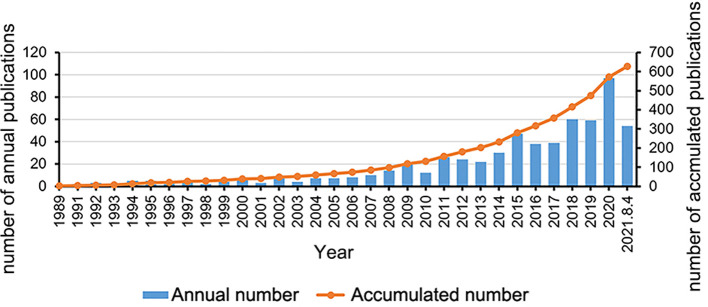
The annual number and accumulated number of publications on meniscal root tears research from 1989 to 2021.

The WOS Core Collection contains 18,331 and 9,257 entries on meniscal and root injuries, respectively, and when the logical algorithm of “and” was performed, there were 626 entries, mainly consisted of 578 articles, 52 reviews, 22 early access articles, letters, etc, and 10 conference proceedings articles. The temporal distribution of the retrieved publications was shown in Figure 1. Based on the analysis of the annual distribution, these years can be divided into two stages, the exploration stage (1989–2007) and the development stage (2008-present): (a) Exploratory phase (1989–2007): In this phase, fewer than 10 papers on meniscal root tears were published each year. During this 18-year period, a total of 84 papers were published. During this period, there was a lack of awareness of the problem of meniscal root tears and of the biomechanical damage caused by MRT, so the treatment of MRT was based on traditional conservative treatment or symptomatic treatment such as rhizotomy. During this period, the entire field was still in an exploratory and experimental phase, and Petersen and Zantop first proposed the use of a tibial tunnel approach to repair meniscal root injuries in 2006 (25), and they introduced the concept of meniscal root repair into the field of research. (b) Development phase (2008-present): in this phase, the total number of papers was 542 by 2021, with a rapid growth in the number of researchers, a high number of publications per year, and a high growth rate. In 2008, Allaire R's team published a biomechanical study of posterior root tears of the medial meniscus, in which they concluded that there was no difference between peak contact pressure after the total medial meniscectomy and that associated with the root tear, both of which result in significant changes in contact pressure and knee joint kinematics, leading to accelerated degenerative changes in the joint, and meniscal root repair can correct joint biomechanics to within normal conditions compared with meniscectomy (26). This is the first article to introduce the concept of “biomechanics” into the field of MRT research, demonstrating the theory that a complete posterior root tear of the medial meniscus would theoretically result in a functional meniscectomy. This article provided a research direction of the relationship between structural damage and biomechanical functional changes, as well as a biomechanical basis for the surgical repair of posterior root tears of the medial meniscus, which led to a dramatic change in the treatment of meniscal root tears. Choi et al. (27) in 2008 proposed a new surgical technique “anchored repair with wires”. This technique has created new possibilities for meniscal root repai, the appearance of these articles has led more and more scholars to devote themselves to research in surgical repair creating more research directions, findings., such as the new FasT-Fix® all-inside suture technique, a modified Mason-Allen Stitch, and the evaluation of different suture techniques from a biomechanical point of view (28–30). The proposal is in line with the clinical situation, and more in-depth research is needed for the improvement of root restoration.

### Distribution of countries and institutions

**Figure 2:**
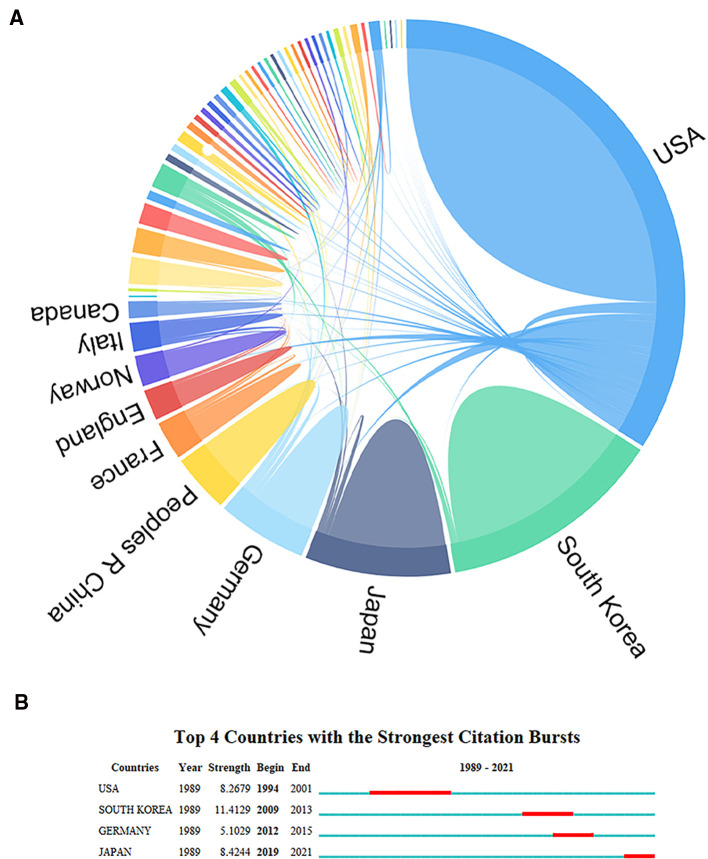
Scientific collaboration network among countries for meniscal root tears research. (**A**) Chord diagram of cooperation network of the publishing countries. (**B**) The strongest citation bursts of countries.

**Figure 3:**
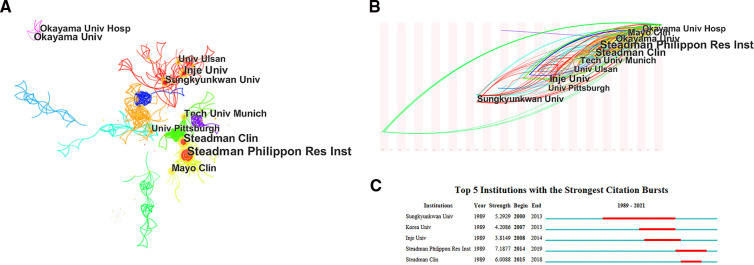
The collaboration network among institutions for eniscal root tears research. (**A**) The clusters of cooperation network of the publishing institutions. (**B**) Time zone diagram of the clusters of cooperation network of the publishing institutions. (**C**) The strongest citation bursts of institutions.

The number of papers published in each country represents the extent of research activity in that country. Of the 626 publications, researchers from 50 countries have contributed to the field thus far. These countries include both developing and developed countries. The specific publications of the top 10 countries in terms of number of publications are shown in Table 1, with the United States (251 publications, 40.10%) and South Korea (113 publications, 18.05%) being the top two countries in terms of number of publications, followed by Japan (74 publications, 11.82%), Germany (51 publications, 8.15%), China (31 publications, 4.95%), and France (23 publications, 3.67%). The collaborative relationships between countries in the field of meniscal root tears were analyzed using social network analysis (Figure 2), and as the top-ranked country, the United States plays a central and important role in the collaborative network and has extensive cooperation with its countries. However, compared with a series of the Occident such as the United States and Germany, cooperation between Asian countries, mainly China, Japan, Korea, and other countries, is less common, which may be due to the differences in the physiological structure/characteristics of the Eastern and Western populations caused by geographical factors. For the institutional analysis, the number of global meniscal root tear papers published involved a total of 764 institutions, and among the top 10 publishing institutions, four institutions were from North America, five were from East Asia, and the remaining institution was from Central Europe (Table 2), which may also be an important reason for the large number of papers published in these regions. On the other hand, it also shows that the formation of top research institutions is an important way to improve a country's academic influence. Among these 10 institutions, **STEADMAN PHILIPPON RES INST** has the highest number of publications (46), the highest H-index (22), and the highest total citations (1,508), while **UNIV PITTSBURGH** ranks sixth in terms of the number of publications, but it has the highest average number of citations (55.13), which indicates that its publications are of high quality. Figure 3 shows the cooperation network of research institutions. A node in the figure represents an institution. From 1989 to 2021, different institutions had network cooperation clusters, as shown in Figure 3A. **STEADMAN PHILIPPON RES INST** is the most productive and influential institution and has extensive cooperation with many other institutions around the world, but, the cooperative relationship between the different institutions shows a clear territoriality: institutions in the same country have a close cooperative relationship with each other, maximizing their locational advantages; compared to the extensive cooperation between countries in Europe and America, there is a lack of obvious cooperative relationship between East Asian countries and European and American countries. Figure 3B shows the time zone diagram of research institutions, from which we can see that the top 10 publishing institutions all appeared after 2000, among which **SUNGKYUNKWAN UNIVERSITY** appeared the earliest and started its research in this field in 2000. This is why there were fewer than 10 publications per year during the discovery phase of the field. Figure 3C shows the top 5 institutions with the strongest citation increase, among which the last three institutions, **SUNGKYUNKWAN UNIVERSITY** (2000–2013), **KOREA UNIVERSITY** (2007–2013), and **INJE UNIVERSITY** (2008–2014), are from Korea, which proves that the research on MRT started early in East Asia, especially in Korea. The research results of **SUNGKYUNKWAN UNIVERSITY** and other institutions are very important to promote the development of the exploratory phase of the field, but probably due to the lack of support from funding agencies for research projects, the United States has gradually occupied the field since 2014.

**Table 1:** The top 10 countries/regions by publications, citations and centrality.

Rank	Country/Region	Publications	% of 626	Total citations	Average citations	H-index
1	USA	251	40.10	7,386	29.43	47
2	SOUTH KOREA	113	18.05	3,259	28.84	34
3	JAPAN	74	11.82	579	7.62	13
4	GERMANY	51	8.15	1,239	24.29	22
5	PEOPLES R CHINA	31	4.95	167	5.39	8
6	FRANCE	23	3.67	205	8.91	7
7	ENGLAND	19	3.04	205	10.79	7
8	NORWAY	16	2.56	201	12.56	7
9	ITALY	15	2.40	228	15.2	5
10	CANADA	13	2.08	518	39.85	7
11	INDIA	13	2.08	150	11.54	4
12	TURKEY	13	2.08	222	17.08	5

**Table 2:** Top 10 institutions distributed by publications and centrality.

Rank	Institution	Publications	% of 626	Original country	Total citations	Average citations	H-index
1	STEADMAN PHILIPPON RES INST	46	7.35	USA	1,508	32.78	22
2	STEADMAN CLIN	37	5.91	USA	1,149	31.05	18
3	INJE UNIV	26	4.15	KOREA	989	38.04	17
4	OKAYAMA UNIV	24	3.83	JAPAN	216	8.64	9
5	MAYO CLIN	22	3.51	USA	311	14.14	9
6	SUNGKYUNKWAN UNIV	20	3.20	KOREA	694	34.7	12
7	TECH UNIV MUNICH	20	3.20	Germany	437	21.85	11
8	OKAYAMA UNIV HOSP	17	2.72	JAPAN	71	4.18	6
9	UNIV ULSAN	17	2.72	KOREA	426	25.06	10
10	UNIV PITTSBURGH	15	2.40	USA	827	55.13	8

There is no doubt that the development of innovative technologies and methods requires significant financial resources, so we obtained information on these research-funding agencies (Table 3), and the main financial supporters are medical device companies and national foundations such as **ARTHREX, SMITH / NEPHEW,NATIONAL INSTITUTES OF HEALTH NIH USA (NIH)** and **UNITED STATES DEPARTMENT OF HEALTH HUMAN SERVICES (HHS)**. The distribution of the top 15 funders is almost entirely from North America. In terms of the distribution of funding agencies, almost all of the top 15 funding agencies are from North America, with the top two being two global medical device companies, **ARTHREX** and **SMITH / NEPHEW**, and **NIH** as a division of **HHS**, they are tied for third. The above results show that developed countries, especially the United States, are absolutely dominant in this field, which is inseparable from adequate financial investment, which allows for the better development of equipment and consumables related to root tear repair surgery. Of course, sufficient funding can also attract more kinds of researchers and institutions to invest more work in this field, which is a mutually reinforcing process. These results indicate that there is extensive cooperation among researchers worldwide, suggesting that research in this field has reached a mature level and that although some institutions and countries have fewer publications, they still have a relatively large number of partners. However, we found a lack of cooperation between East Asian countries and European and American countries in this field, and there seems to be insufficient cooperation between top institutions. These situations may be because, first, factors such as geography, living conditions and habits lead to differences in the physiological structure as well as physiological characteristics of different human races (8–10), and thus, the focus rooms of the research institutes in the two regions are different, and second, the cutting-edge research power is relatively mature. They have clear research directions and independent research strengths, and it seems unnecessary to actively seek more opportunities for collaboration.

**Table 3:** The top 10 related funding agencies.

Rank	Funding agencies	Countries/ Regions	Publications	% of 626
1	ARTHREX	USA	21	3.36
2	SMITH NEPHEW	USA	16	2.56
3	NATIONAL INSTITUTES OF HEALTH NIH USA	USA	13	2.08
4	UNITED STATES DEPARTMENT OF HEALTH HUMAN SERVICES	USA	13	2.08
5	NIH NATIONAL INSTITUTE OF ARTHRITIS MUSCULOSKELETAL SKIN DISEASES NIAMS	USA	9	1.44
6	NATIONAL NATURAL SCIENCE FOUNDATION OF CHINA NSFC	CHINA	8	1.28
7	HISTOGENICS	USA	7	1.12
8	STEADMAN PHILIPPON RESEARCH INSTITUTE	USA	7	1.12
9	STRYKER	USA	7	1.12
10	ARTHRITIS FOUNDATION	USA	6	0.96
11	BIOMET	USA	6	0.96
12	CETERIX	USA	6	0.96
13	GLAXOSMITHKLINE	UKA	6	0.96
14	NOVARTIS	SWIT	6	0.96
15	PFIZER	USA	6	0.96

### Distribution of authors

**Figure 4:**
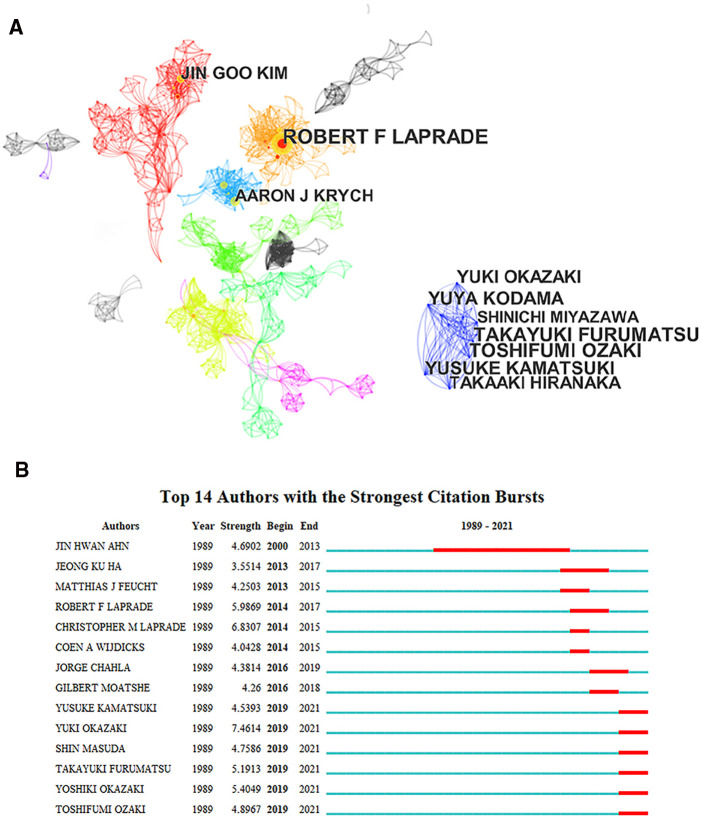
The collaboration network among authors for tear of meniscal root tears research. (**A**) The clusters of cooperation network of authors (**B**) The strongest citation bursts of authors.

The number of scientific papers published by the authors represents, to some extent, the authors’ contribution and activity in this field. The 626 articles on meniscal root tears were drafted by approximately 2,053 authors, although most of the authors have published only one or two papers, they have contributed to the development of research in this field. Table 4 lists the top 10 most published authors, who are professional and active writers in this field. Among these authors, except for LAPRADE RF and KRYCH AJ from the United States, the rest are from East Asian countries. This result indicates that East Asian countries have a prominent contribution in the field of MRT and explains that despite the lack of support from a large number of funding agencies, Japan and Korea are still second only to the United States in the overall research field. However, from the analysis of author collaboration (Figure 4A), the centrality index of each author is <0.1, and rather few linkages are observed in this network diagram, reflecting that there is little collaboration research teams from different countries, especially between East Asia and Europe and America. The main reason for this phenomenon may be that MMPRTs caused by degenerative injury are the most common compared to LMPRTs caused by trauma, and a lifestyle of prolonged squatting and kneeling, cross-legged sitting, etc, is a very common influence on MMRPTs (9), this lifestyle is particularly common in Japan and Korea, so authors and research institutions in these countries have a large number of samples to draw from. This explains the lack of collaborative relationships between East Asian countries and European and American countries, which may be due to the different focus of the studies. In addition, our results show that LAPRADE RF is the author with the highest number of publications and the most cited author, with a focus on anatomy and biomechanics and a morphological-based typing of MRT tears, who has made a remarkable contribution to the subsequent guidance of surgical treatment (4). Figure 4B shows the strongest citation bursts of authors, whose number of publications in this field increased rapidly over a certain period of time, and among these 14 authors, AHN JH (2000–2013) is the only one who has published extensively since the exploratory phase of the field, leading his team to design the “Posterior trans-septal portal”. With this portal, complete arthroscopic visualization of the posterior compartment and easier arthroscopic procedures for the posterior compartment of the knee joints are possible (31), and a new pull-out suture for transection of the posterior horn of the medial meniscus was also derived with the aid of this portal (32). The above results show that although there have been collaborative relationships between authors, such collaborations exist only within the same country or even within the same research institution. Therefore, more attention should be paid to exchanges between different countries and even some cross-disciplinary collaborations to improve the development of the field. The above findings may also help researchers to identify active research groups in the field and provide a reference for them to seek potential academic collaborations.

**Table 4:** Top 10 authors distributed by publications and citations.

Rank	Author	Publications	% of 626	Institution	Original country	Total citations	Average citations	H-index
1	LAPRADE RF	47	7.51	STEADMAN PHILIPPON RES INST	USA	1,548	32.94	22
2	FURUMATSU T	39	6.07	OKAYAMA UNIV	JAPAN	272	6.97	9
3	OZAKI T	38	5.91	OKAYAMA UNIV	JAPAN	272	7.16	9
4	OKAZAKI Y	32	4.95	OKAYAMA UNIV	JAPAN	153	4.78	7
5	KAMATSUKI Y	31	4.79	OKAYAMA UNIV	JAPAN	199	6.42	8
6	KODAMA Y	31	4.79	OKAYAMA UNIV	JAPAN	257	8.29	9
7	HIRANAKA T	26	3.99	OKAYAMA UNIV	JAPAN	82	3.15	6
8	KIM JG	25	3.99	INJE UNIV	KOREA	824	32.96	14
9	KRYCH AJ	22	3.51	MAYO CLIN	USA	311	14.14	9
10	MIYAZAWA S	20	3.20	OKAYAMA UNIV	JAPAN	180	9	8

### Distribution of journals

Analysis of source journals and research topics in a research field can help researchers accurately grasp the direction of development of the research field and core journals and provide professional guidance for literature searches, data collection, paper writing and submission. A total of 626 SCI-related meniscal root tear research articles were published from 1989 to 2021. The top 11 journals with the most published articles are listed in Table 5, and the total number of papers published in these 11 journals accounted for more than 55% of the total number of included papers. It is easy to see that the journals listed in Table 5 are mainly related to surgery, orthopedics, sports medicine and radiology, nuclear medicine / medical imaging; in addition, all of these journals are from Europe and the United States, which is further evidence of the dominance of Europe and the United States in this field. We found that most output journals were categorized as Q1 or Q3, with AMERICAN JOURNAL OF SPORTS MEDICINE (IF6.202, Q1) having the highest IF and KNEE SURGERY SPORTS TRAUMATOLOGY ARTHROSCOPY (IF4.342, Q1) was the most productive journal. This indicates that the high output as well as the high quality of research contribute to the academic impact of the journals. Among these journals, AM J SPORT MED and ARTHROSCOPY are the top journals in the field, and the articles published in them influence the direction of research in the field as a whole, because the articles published in these journals represent the most advanced technologies and theories in the discipline or focus on the most controversial scientific issues in the field, which open up new research ideas for subsequent scholars and influence the direction of the field. Influencing the direction of the field. For example, an article by Marzo JM published in AM J SPORT MED in 2009 demonstrated in cadaveric specimens that posterior horn medial meniscal root avulsion leads to deleterious alteration of the loading profiles of the medial joint compartment, resulting in a significant increase in peak medial joint contact pressure and a significant decrease in contact area. However, surgical repair restores the ability of the medial meniscus to absorb hoop stress and eliminate joint space narrowing, restoring the loading profiles to values equal to the control knee, possibly decreasing the risk of degenerative disease (33). This article joins Allaire R's article in J Bone Joint Surg Am in providing a biomechanical rationale for meniscus root repair surgery, and the increasing recognition by physicians of the importance of preserving the meniscus as much as possible to prevent cartilage degeneration and overall osteoarthritis in the knee. This change in treatment philosophy has led to a shift from targeting meniscectomy for pain resolution, faster recovery and avoidance of reoperation to a focus on restoring the meniscus to its original physiologic function and thus avoiding a range of long-term adverse outcomes. The aforementioned journals are core journals that are extremely popular among researchers in this field, and in the future, more relevant findings will be published in these journals.

**Table 5:** The top 10 journals distributed by publications.

Rank	Journal	Publications	% of 626	IF (JCR 2020)	JIF quartile
1	KNEE SURGERY SPORTS TRAUMATOLOGY ARTHROSCOPY	99	15.82	4.342	Q1
2	AMERICAN JOURNAL OF SPORTS MEDICINE	61	9.74	6.202	Q1
3	ARTHROSCOPY THE JOURNAL OF ARTHROSCOPIC AND RELATED SURGERY	52	8.31	4.772	Q1
4	ARTHROSCOPY TECHNIQUES	38	6.07		–
5	KNEE	27	4.31	2.199	Q3
6	SKELETAL RADIOLOGY	23	3.67	2.199	Q3
7	ORTHOPAEDIC JOURNAL OF SPORTS MEDICINE	18	2.88	2.727	Q2
8	ORTHOPAEDICS TRAUMATOLOGY SURGERY RESEARCH	18	2.88	2.256	Q3
9	ARCHIVES OF ORTHOPAEDIC AND TRAUMA SURGERY	15	2.40	3.067	Q2
10	AMERICAN JOURNAL OF ROENTGENOLOGY	13	2.08	3.959	Q2
11	JOURNAL OF KNEE SURGERY	12	1.92	2.757	Q3

**Figure 5:**
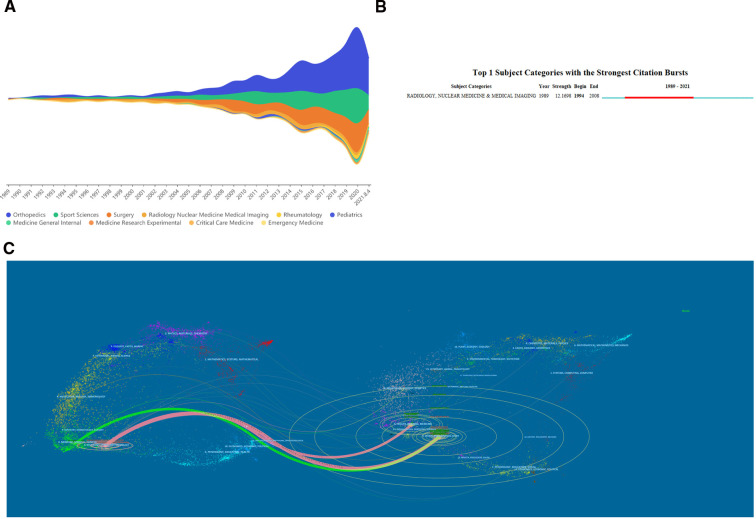
The discipline wise contribution of meniscal root tears research. (**A**) Steam graph of the top 10 research areas. (**B**) The strongest citation bursts of research categories. (**C**) The dual-map overlay of journals related to meniscal root tears research.

Figure 5A is a density visualization summarizing the top 10 disciplines between 1989 and 2021. Orthopedics, sports medicine and surgery are the three main disciplines that are popular for research, and research in these three areas has continued to grow rapidly in recent years. Figure 5A is a density visualization summarizing the top 10 disciplines between 1989 and 2021. Orthopedics, sports medicine and surgery are the three main disciplines that are popular for research, because meniscal root tear is a routine orthopaedic condition, as well as a sports injury-related condition, the growing momentum of meniscal root research and the popularization of arthroscopy technique in the past decades, making meniscus surgery a common treatment, which has led to the continued development of three areas of research. Figure 5B shows that a large number of studies in radiology, nuclear medicine / medical imaging emerged between 1994 and 2008, and this emergence may be closely related to the development and popularity of MRI. We conducted a dual-map overlay of journals (Figure 5C). The graph shows the disciplines covered by the journals in the form of labels, and the colored line segments in the double graph indicate the cited connections, which trace the cited article back to the cited journal. The studies published in Medicine/Medical/Clinical/Sports/Surgery journals primarily cite research published in Health/Nursing/Medicine,Sports/Rehabilitation/Surgery and Molecular/Biology/Genetics, more information on representative citations and classified journals in each category can be found in Figure 5C. For example, the most representative journals in the Sports/Rehabilitation/Surgery classification are The American Journal of Sports Medicine, Arthroscopy, Knee Surgery Sports Traumatology Arthroscopy, and Journal of Bone and Joint Surgery. These results are mainly due to the fact that MRT is a common clinical condition, and the mechanism of injury, clinical diagnosis, ancillary tests, treatment modalities, and prognosis associated with this condition are the basis of the entire field of research. The above results indicate that the research literature published in field journals is mainly focused on the diagnosis, clinical treatment and postoperative rehabilitation of different types of sports injuries and demonstrate that the restoration of the original biomechanical and kinematic functions of root tears after different treatments and rehabilitation is still the main direction of current research.

### Reference co-citation analysis

References are an important part of a high-quality paper, not only providing a strong argument for the author's findings but also expanding the information chain and reflecting the scientific value of the research. The number of citations is a common evaluation metric used in scientometric analysis to quantify the relative influence of scientific articles in a given subject area, as the influence and recognition of an article is usually proportional to the number of citations to that article (34). Highly cited literature is generally defined as high-quality research that has a high impact in terms of innovation and discovery in a particular field. That is, these publications are also considered essential for scholars preparing for this work. Table 6 shows the details of the top 10 most highly cited original articles within the Half Moon Root Tear study, which were published between 2004 and 2015 (Table 6), with three of them published before 2008. In general, modularity *Q* and subject contour value *S* are two indicators used to evaluate clustering. *Q* > 0.3 indicates that the network is very important, and >0.5 indicates that the clustering results are reasonable; clustering is reasonable when the contour value (*s*) is >0.5 and convincing when *s* is >0.7 (35). In our study, *Q* = 0.676, which is greater than 0.5, which means that the network is reasonably divided into loosely coupled clusters and that only clusters #0 and #4 out of the first 10 clusters have profile values below 0.7 (Table 7), which indicates that this cluster is convincing. The most cited reference in our dataset is a 2008 study by Allaire et al. on the biomechanics of posterior root tears of the medial meniscus, which has over 250 co-citations. As mentioned above, Allaire R pioneered the study of the biomechanics of meniscal root injuries and provided the theoretical basis for the development of surgical repair of posterior root tears of the medial meniscus.

**Table 6:** The top10 references with most co-citation counts in publications on tear of the root of the meniscus.

Rank	Co-citation counts	Cited reference	Representative author (publication year)	Journal	DOI
1	256	Biomechanical consequences of a tear of the posterior root of the medial meniscus. Similar to total meniscectomy	Allaire R, 2008	J Bone Joint Surg Am, v90a, p1922	10.2106/jbjs.g.00748
2	132	Meniscal root tears: significance, diagnosis, and treatment	Bhatia S, 2014	Am J Sport Med, v42, p3016	10.1177/0363546514524162
3	125	The role of meniscal root pathology and radial meniscal tear in medial meniscal extrusion	Lerer DB, 2004	Skeletal Radiol, v33, p569	10.1007/s00256-004-0791-2
4	122	Effects of medial meniscus posterior horn avulsion and repair on tibiofemoral contact area and peak contact pressure with clinical implications	Marzo JM, 2009	Am J Sport Med, v37, p124	10.1177/0363546508323254
5	113	Medial meniscus root tear refixation: comparison of clinical, radiologic, and arthroscopic findings with medial meniscectomy	Kim SB, 2011	Arthroscopy, v27, p346	10.1016/j.arthro.2010.08.005
6	108	Radial tears of the posterior horn of the medial meniscus	Bin SI, 2004	Arthroscopy, v20, p373	10.1016/j.arthro.2004.01.004
7	97	Radial tears in the root of the posterior horn of the medial meniscus	Ozkoc G, 2008	Knee Surg Sport Traumatol Arthrosc, v16, p849	10.1007/s00167-008-0569-z
8	93	Medial meniscus extrusion on knee MRI: is extent associated with severity of degeneration or type of tear?	Costa CR, 2004	Am J Roentgenol, v183, p17	10.2214/ajr.183.1.1830017
9	90	Meniscal root tears: a classification system based on tear morphology	Laprade CM, 2015	Am J Sport Med, v43, p363	10.1177/0363546514559684
10	89	Arthroscopic suture anchor repair versus pullout suture repair in posterior root tear of the medial meniscus: a prospective comparison study	Kim JH, 2011	Arthroscopy, v27, p1644	10.1016/j.arthro.2011.06.033

**Table 7:** The significant clusters of co-citation of reference.

Cluster ID	Cluster label (LLR)	Size	Silhouette value	Mean year	Leading document
0	Anterior cruciate ligament	91	0.684	2008	Allaire R, 2008, J Bone Joint Surg Am, v90A, p1922
1	Transtibial pullout repair	55	0.743	2013	Bhatia S, 2014, Am J Sport Med, v42, p3016
2	Posterior horn	48	0.777	1996	Brody JM, 2006, Radiology, v239, p805
3	Lateral meniscus	43	0.9	1999	Ahn JH, 2009, Knee, v16, 77
4	Meniscus luxation	41	0.692	1999	Costa CR, 2004, Am J Roentgenol, v183, p17
5	Knee ligaments menisci and cartilage	38	0.953	1973	Goldman AB, 1988, Am J Roentgenol,v 151, p1163
6	Intercondylar eminence	30	0.981	1994	Meyers MH, 1970, J Bone Joint SURG AM, vA 52, p1677
7	Avulsion fracture	14	0.998	1985	Ogden JA, 1980, J Bone Joint SURG AM, v62, p205
8	3t	11	0.973	2001	Kijowski R, 2009, Radiology, v252, p486

**Figure 6:**
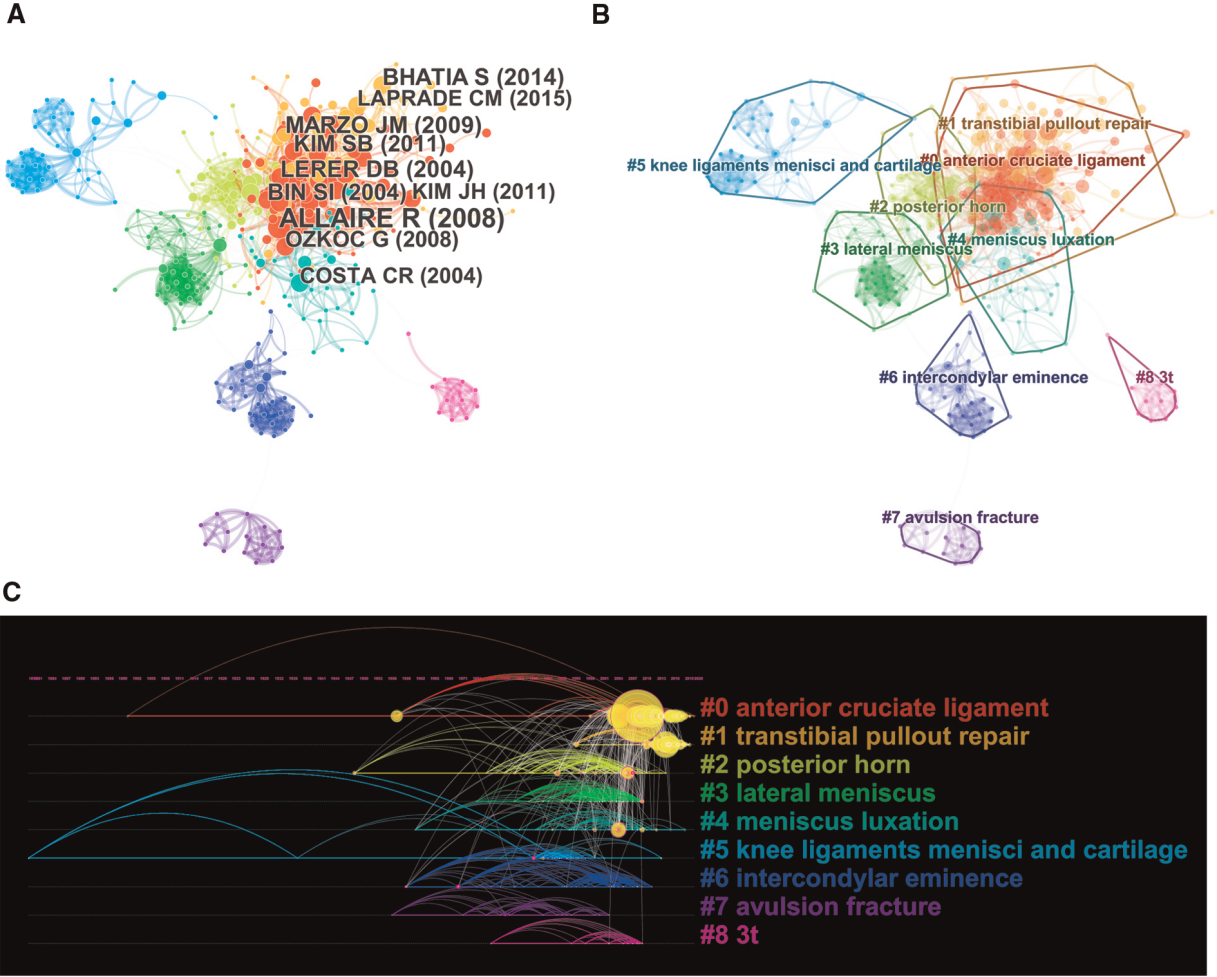
The co-citation analysis of references in publications on meniscal root tears research. (**A**) Co-citation network of references. (**B**) Cluster map of co-citation network of references. (**C**) The timeline view of co-citation network of references.

**Figure 7:**
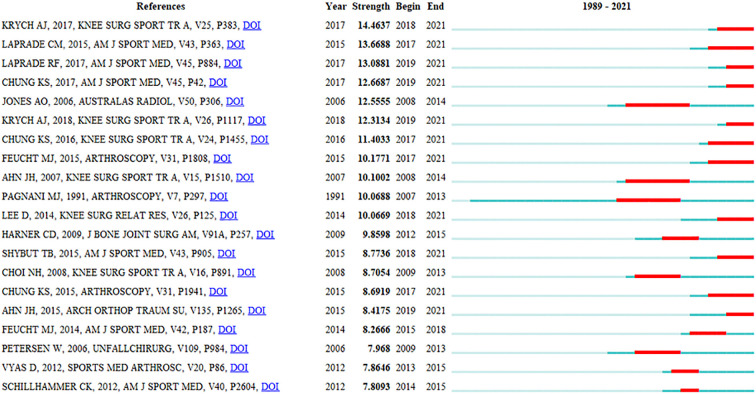
Top 20 references with the strongest citation bursts in publications on meniscal root tears research.

In citation analysis, when the effect of time as a confounding factor must be considered, co-citation maps and cluster maps can be obtained for the cited literature (Figures 6A,B). Figure 6B gives the largest 9 clusters in the reference co-citation network based on the log-likelihood ratio algorithm in CiteSpace software, and the clusters where each citation is located are arranged by time to obtain the citation timeline map (Figure 6C), as well as the emergent literature (Figure 7). Among these clusters, Clusters #2–#8 first appeared during the developmental phase (1989–2007), during which research on meniscal root tears focused on the understanding of the dissected structures, the diagnosis and identification of the disease, and the adverse consequences associated with structural damage, while a large body of literature on radiology and medical imaging emerged during this period to lay a solid foundation for the noninvasive diagnosis of the disease. In terms of treatment, early meniscal root injuries, because they are difficult to diagnose and identify, are treated mostly by conservative treatment or meniscectomy, which may initially improve symptoms and functional knee scores in patients with meniscal tears, but these treatments do not restore the natural meniscal anatomy and function and may lead to joint space narrowing and arthritic changes over time. Alford Lerer (36) by evaluating MRI in 205 patients subsequently, their study demonstrated that there is a significant association between pathologic medial meniscal extrusion (MME) and degenerative joint disease (DJD), MMR pathology and radial tears, and that MME may precede the development of medial femoral-tibial DJD as an etiology rather than a consequence of DJD. Lerer demonstrated for the first time the relationship between MMR and MME and DJD, namely that MMR pathology disrupts the ability of the meniscus to withstand cyclic stresses thereby leading to pathological MME, which over time progresses to DJD. The proposed relationship, while questioning traditional treatment modalities, inspired the possible need to develop techniques to repair meniscal root injuries arthroscopically. Petersen (25) first proposed the use of a tibial tunnel technique to repair meniscal root injuries in 2006, the main method of which consisted of drilling a tibial tunnel in the area of the tibial attachment at the posterior root of the medial meniscus and pulling the meniscal root out of the osseous tract, which were then secured to the distal end of the tibial tunnel. This technique of repairing meniscal root tears through the tibial tunnel pioneered a completely new surgical approach to the possibility of repairing meniscal root injuries, a technique that preserves the integrity of the meniscus, approximates the restoration of the biomechanics of the meniscus, and thus prevents a series of adverse outcomes resulting from root injuries, a milestone in meniscal root treatment techniques.

Clusters #0 and #1 appeared mainly after 2007 (developmental phase), during which a great deal of research was conducted on the function of the meniscus and meniscal root biomechanics, while with the development of arthroscopic techniques, meniscal root repair techniques were more intensively studied, and more attention began to be paid to surgical complications, long-term surgical efficacy, factors affecting surgical efficacy, and the variability of different surgical techniques (29). Choi (27) first proposed fixation of a metal suture anchor with two fiber wires to the root of the torn meniscus and knot fixation on the surface of the meniscus as an alternative to meniscectomy. Mao et al. (37) described two sutures that can be used for paracentral radial tears (types I, II, and IV) where there is a significant stump at the root attachment point and the meniscus needs to have good and more complete morphology at both ends of the tear. The advent of these surgical techniques, while laying the groundwork for repair techniques, has provided more possibilities for surgical techniques and inspired more scholars to begin research on injury repair, with one study showing a 37% increase in meniscal repair rates from 2004 to 2012 (38), and has broadened the criteria and indications for meniscal repair, setting off a wave of research on injury repair.

“Anterior cruciate ligament” (Cluster #0) and “transtibial pullout repair” (Cluster #1) are the two largest clusters with a total of 146 publications since the field entered the development phase compared to the decreasing attention given to Clusters #2–#8. The two clusters have received increasing attention from researchers (Figure 6C), which shows that the literature on root restoration techniques and comparative clinical efficacy studies of surgical approaches are still the main research hotspots. Figure 7 shows the top 20 references highlighted by the cited literature, and although the 10 most cited articles shown in Figure 6A are considered landmark articles due to the importance of their contributions, there is a moderate correlation between the year of publication and the number of citations, i.e., the earlier an article is published, the more it is likely to be cited (39). Therefore, some of the most recently published high-quality research cannot be determined by citation counts alone. To track and capture the evolution of research hotspots, literature bursts were analyzed using CiteSpace. In general, articles with citation bursts imply that they have received special attention from the relevant academic community in the past period (40).

### Keyword co-citation analysis

**Figure 8:**
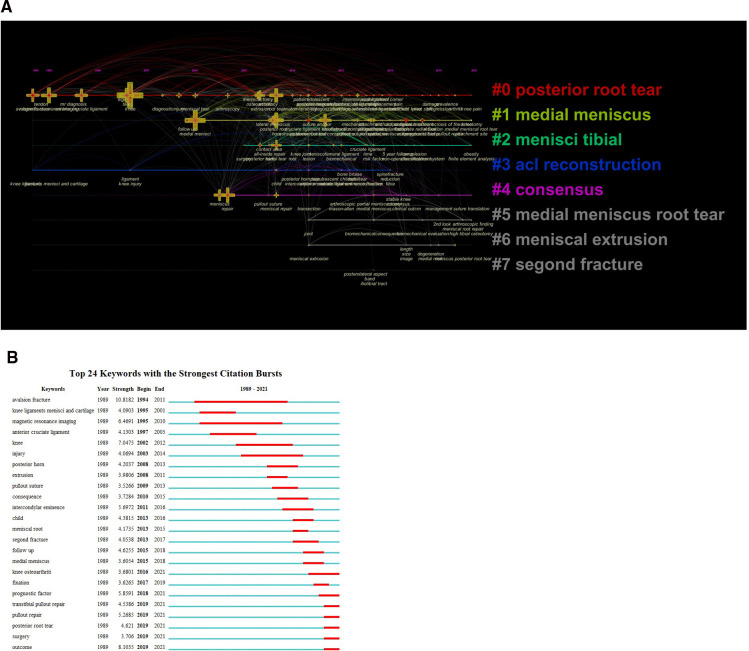
The co-occurrence network and clusters of keywords in publications on meniscal root tears research. (**A**) The timeline view of cluster map of co-occurrence network of keywords (**B**) The strongest citation bursts of keywords.

Keywords are usually standardized representative terms used to express the topic of a paper, and the statistical analysis of keywords on the topic of literature provides insight into the research hotspots and future research directions in the field. The authors generally agree that the more frequently the terms appear in the same literary work, the closer the relationship between these two topics; keywords with high centrality play an important role as a link and medium in the keyword network map, and the more obvious the centrality is, the stronger the control and guidance of the whole network, indicating that the keywords are highly concerned. As shown in Table 8, the keywords with a high frequency of use were “meniscus”, “knee”, “medial meniscus”, “root tear” and “anterior cruciate ligament”, and the keywords with high centrality were “meniscal tear”, “avulsion fracture”, “extrusion”, “intercondylar eminence” and “anterior cruciate ligament”. Studies published from 1989 to 2021 were selected for CiteSpace analysis for time slices plotting keyword timelines, and Table 9 shows that the high-frequency items related to meniscal root tear research were clustered into 9 main categories, and the top 5 included Cluster #0, posterior root tear; Cluster #1, medial meniscus; Cluster #2, menisci tibial; Cluster #3, ACL reconstruction; and Cluster #4, consensus. In recent years, few new keywords have appeared, suggesting that the field of meniscal root tears has made fewer breakthroughs in identifying new problems and needs more research accumulation and innovation. The timeline graph (Figure 8A) shows the keyword information contained in these clusters, and the keywords with high frequency in the last 3 years are “attachment site”, “refixation”, “suture repair”, “progression”, “transtibial pullout repair”, etc. This indicates that studies related to repair outcomes are still the focus of treatment attention. Ro KH (41) systematically reviewed articles related to clinical and radiological outcomes of meniscal repair and meniscectomy, including a total of 13 studies, and came to the final conclusion of better MMRT outcomes, greater improvement in Lysholm scores, lower rates of knee osteoarthritis progression, and lower rates of reoperation after meniscal repair compared to partial meniscectomy.

**Table 8:** The top 10 author keywords by frequency and centrality.

Rank	Keywords	Frequency	Rank	Keywords	Centrality
1	Meniscus	89	1	Meniscal tear	0.18
2	Knee	84	2	Avulsion fracture	0.17
3	Medial meniscus	76	3	Extrusion	0.13
4	Root tear	56	4	Intercondylar eminence	0.13
5	Anterior cruciate ligament	55	5	Anterior cruciate ligament	0.12
6	Magnetic resonance imaging	45	6	Repair	0.11
7	Posterior root tear	40	7	Lateral meniscus	0.11
8	Arthroscopy	33	8	Posterior root tear	0.09
9	Lateral meniscus	32	9	Posterior horn	0.09
10	Meniscal repair	30	10	Cruciate ligament reconstruction	0.09

**Table 9:** The significant co-occurrence clusters of keywords in publications on tear of the root of the meniscus.

Cluster ID	Size	Sihouette	Mean (year)	Top terms (log-likelihood ratio, *p*-level)
0	51	0.691	2010	Posterior root tear (6.52, 0.05)
1	46	0.631	2014	Medial meniscus (11.3, 0.001)
2	27	0.647	2013	Menisci tibial (8.13, 0.005)
3	20	0.801	2010	ACL reconstruction (10.14, 0.005)
4	14	0.895	2013	Consensus (10.07, 0.005)
5	9	0.914	2018	Medial meniscus root tear (10.37, 0.005)
6	8	0.893	2017	Meniscal extrusion (13.39, 0.001)
7	3	0.99	2015	Segond fracture (14.24, 0.001)

The frequent occurrence of keywords over time is considered a sign of cutting-edge topics and flourishing and emerging trends. Twenty-four keywords with citation bursts were detected through CiteSpace's analysis of the strongest citation bursts from 1989 to 2021 (Figure 8B), with the keyword “avulsion fracture”, which only ended in 2011, having the highest citation rate (10.82), while posterior root tear (2019–2021), pullout repair (2019–2021), outcomes (2019–2021) and surgery (2019–2021) appeared to be the new hotspots of research in the field. Similar to the above results, the evolution of the strongest citation bursts of keywords in the last three decades is consistent with the whole process of a disease from its development to its treatment and recovery, i.e., the research focus has undergone a shift from the study of anatomical knots and injury mechanisms to the study of treatment methods and prognosis. With the above keyword analysis, all clustering terms in relation to root tears then focus on the developmental phase of the study (2008-present). In this phase, scholars study the biomechanical properties of the meniscus and root in normal/abnormal conditions based on deconvolution science and continue to compare and innovate therapeutic tools.

Jian-Yu Wang et al. (42) used finite element analysis to compare the biomechanical effects of different repair methods on LMPRTs of the knee, and they concluded that the cartilage contact area at the attachment point was essentially restored to normal with the double-stitch technique compared to the single-stitch suture, with contact areas at the medial and lateral gaps of 568.007 and 508.678 mm^2^ (532.254 and 512.286 mm^2^ for the medial and lateral gaps, respectively, when the meniscus is structurally intact). Yan-Song Qi et al. (43) studied changes in knee stability in three conditions (LMPR was intact, LMPR was cut off from its tibial end, and LMPRT was repaired), and they found that LMPRTs can lead to notable internal rotational instability at knee flexion from 30° to 90°. LMPRT repair helped improve internal rotation stability at 30° of knee flexion and 60°–90° and improved anterior stability at 30° of knee flexion and recovery at 60°. Xin Tang et al. (44) studied the effects of lateral posterior meniscal root tears, partial meniscectomy and total meniscectomy on knee biomechanics in ACL reconstruction, and they found that partial meniscectomy had no significant effect on the stability of lateral posterior meniscal root tears in ACL-reconstructed knees under anterior tibial and simulated pivot shift loading. Therefore, clinically, partial meniscectomy may be considered in the setting of ACL reconstruction in cases of irreparable meniscal root tears or persistent pain. Faucett et al. (45) compared meniscal repair, meniscectomy and nonsurgical treatment methods in terms of osteoarthritis progression, total knee replacement rates (clinical effectiveness) and cost-effectiveness, and they concluded that repair was cost-effective or advantageous compared to meniscectomy and nonsurgical treatment from 5 years after surgery.

These findings further illustrate the research trend in the field that the study of meniscal root injury is no longer limited to the biomechanical changes of the meniscus; rather, scholars are paying more attention to whether this injury affects the motor function of the entire knee joint, and for the treatment of MRTs, researchers are now comparing the advantages of different treatment options, their economic effects, and the need to design individualized treatment plans for different patients with the primary goal of solving the patient's pain. These research topics have received a great deal of attention in recent years, so we can expect to see future work dissecting these themes, leading to more interesting scientific discoveries.

### Limitations

This study has some limitations that need to be noted and addressed. (a) This study is based on a single database from WoSCC, which means we may have missed some relevant publications from other databases. However, WoSCC is an authoritative, comprehensive and multidisciplinary core journal citation index database. (b) Our study focused only on English articles, which may reduce the number of retrieved articles. (c) In terms of research methods, we only used classical methods, such as co-citation analysis, cluster analysis from timezone view to analyze the retrieved literature. there are many other methods including bibliographic coupling analysis to conduct such studies. (d) Data generated from articles published after August 2021 were not included in our scientometric analysis because the database is continuously updated and this year's dataset is incomplete.

## Conclusion

The aim of this study is to systematically summarize the knowledge structure and research frontiers of meniscal root tears by means of scientometric and visual analysis methods, providing the latest advances and future perspectives. The current findings clearly indicate that research on meniscal root tear repair techniques continues to be a major part of research in recent years. The most productive countries, institutions, journals and authors are the **USA, STEADMAN PHILIPPON, *KNEE SURGERY SPORTS TRAUMATOLOGY ARTHROSCOPY***, and **LAPRADE RF**. The most frequently cited literature was published in ***JOURNAL OF ANATOMY*** in 2013: “Anatomy of the anterolateral ligament of the knee” was cited 494 times. The results of research in these foundations of health/nursing/medicine and sports/rehabilitation/sport are mostly published in journals related to clinical aspects of medicine such as neurology, kinesiology and ophthalmology. The analysis of keywords, citations, and the corresponding emergence of the literature shows that the current research has shifted from the exploratory phase of basic research on the structure of the dissection and diagnosis of the disease to the developmental phase regarding the biomechanical function of the meniscus and the improvement of surgical repair techniques.

The results of this study will enable scholars who are new to the field to better understand the established research directions, the most influential research groups, and authoritative journals and periodicals, to identify potential research frontiers through keyword emergence, and to focus on making breakthroughs in these research directions, which may lead to significant The research results are likely to produce significant research results, thus greatly promoting the development of the field and providing references for future research directions and scientific decisions. At present, the field is mainly focused on clinical research, and there is less basic research on the treatment of meniscal roots. In the future, more relevant research needs to be attempted: (1) material improvement and technical innovation. The improvement of structural function depends largely on postoperative rehabilitation; (2) cell biological and pathophysiological research, etc.
